# EXPLORING SUBJECTIVE COGNITIVE DECLINE IN OLDER ADULTS: A MIXED METHODS APPROACH

**DOI:** 10.1093/geroni/igae098.4367

**Published:** 2024-12-31

**Authors:** Xi Wen, Huiling Li

**Affiliations:** Soochow University--Suzhou, Suzhou, Jiangsu, China (People’s Republic); Soochow University--Suzhou, Suzhou, Jiangsu, China (People’s Republic)

## Abstract

**Objective:**

This study aims to explore the cognitive health of older adults exhibiting SCD by using a mixed methods approach to identify the factors and implications of perceived cognitive decline, thereby contributing to early intervention strategies.

**Methods:**

We employed a convergent parallel mixed-method design. Quantitative data were collected using the Dementia Assessment Sheet for Community-based Integrated Care System 21 items (DASC-21) and the 9-items Subjective Cognitive Decline Questionnaire (SCD-Q9) from 1,458 older adults without clinical dementia in Soochow, China. Qualitative data were gathered through semi-structured interviews to capture detailed narratives on cognitive complaints, their perceived causes, and impacts on daily life.

**Results:**

Quantitative analysis revealed that 48.7% of participants experienced SCD, with significant associations found with advanced age, low education levels, underweight, diabetes, cataract/glaucoma, osteoporosis, hyperlipidemia, and ischemic heart disease. Qualitative thematic analysis identified three primary themes: cognitive complaints, multifactorial attributions of cognitive decline, and the effects on daily life.

**Conclusion:**

Our findings underscore the importance of recognizing SCD in older adults as a possible precursor to more severe cognitive impairment. The mixed methods approach provided a comprehensive understanding, suggesting that both objective and subjective assessments are critical for early detection and intervention. These insights highlight the need for tailored interventions focusing on modifiable risk factors and enhancing quality of life for older adults with SCD.

